# Celiac disease and hepatitis C relationships in transcriptional regulatory networks 

**Published:** 2017

**Authors:** Fereshteh Izadi, Mostafa Rezaei Tavirani, Zahra Honarkar, Mohammad Rostami-Nejad

**Affiliations:** 1 *Basic and Molecular Epidemiology of Gastrointestinal Disorders Research Center, Research Institute for Gastroenterology and Liver Diseases, Shahid Beheshti University of Medical Sciences, Tehran, Iran*; 2 *Proteomics Research Center, Shahid beheshti University of Medical Sciences, Tehran, Iran*; 3 *Gastroenterology Unit, Modares Hospital, Shahid beheshti University of Medical Sciences, Tehran, Iran *; 4 *Gastroenterology Department, Atiyeh Hospital, Tehran, Iran*; 5 *Gastroenterology and Liver Diseases Research Center, Research Institute for Gastroenterology and Liver Diseases, Shahid Beheshti University of Medical Sciences, Tehran, Iran *

**Keywords:** celiac disease, hepatitis c, regulatory network

## Abstract

**Aim:**

we mainly aimed to elucidate potential comorbidities between celiac disease and hepatitis c by means of data and network analysis approaches.

**Background:**

understanding the association among the disorders evidently has important impact on the diagnosis and therapeutic approaches. Celiac disease is the most challenging, common types of autoimmune disorders. On the other hand, hepatitis c virus genome products like some proteins are supposed to be resemble to gliadin types that in turn activates gluten intolerance in people with inclined to gluten susceptibilities. Moreover, a firm support of association between chronic hepatitis and celiac disease remains largely unclear. Henceforth exploring cross-talk among these diseases will apparently lead to the promising discoveries concerning important genes and regulators.

**Methods:**

321 and 1032 genes associated with celiac disease and hepatitis c retrieved from DisGeNET were subjected to build a gene regulatory network. Afterward a network-driven integrative analysis was performed to exploring prognosticates genes and related pathways.

**Results:**

105 common genes between these diseases included 11 transcription factors were identified as hallmark molecules where by further screening enriched in biological GO terms and pathways chiefly in immune systems and signaling pathways such as chemokines, cytokines and interleukins.

**Conclusion:**

in silico data analysis approaches indicated that the identified selected combinations of genes covered a wide range of known functions triggering the inflammation implicated in these diseases.

## Introduction

 Celiac disease (CD), as a chronic immune-mediated disorder, is caused by gluten ingestion that exhibits a wide spectrum of clinical representations, including intestinal abnormalities and malabsorption (1). CD is a genetic based disease with a 1% worldwide prevalence especially in western part of the world. Variety of manifestations like gut lesions are triggered by environment (gluten, infection, etc.) and genetic (HLA and non-HLA genes). CD is further characterized by severe autoimmune responses resulting in damaging small intestinal mucosa (1, 2). On the other hand, the hepatitis C virus (HCV) is a serious health burden which infects 170 million people globally that mostly asymptomatic in nature (3). Chronically infected patient’s present liver injury including liver cirrhosis and liver failure (4). The remarkable ability of the virus to establish persistent infections (5-8), in addition to the poor understanding in pathogenesis of HCV, demands in-depth studies in elucidating HCV complex biological processes. Of note, being infected with HCV in long-term definitely increases the risk of liver cancers such as hepatocellular carcinoma (HCC) (4). Although the most of CD patients indicated different gastrointestinal anomalies, they are also affected by several complications (9-12). Moreover several hepato- anomalies including cholangitis (14), hepatic T-cell lymphoma (14) as well as pancreatitis resulting in acute changes in endocrine system have been documented in symptomatic CD patients (14). CD-related changes in liver functions are thought to be due to the production of some agents like reactive oxygen species, tumor necrosis factor-α (TNF-α) and inflammatory responses. 

The complex molecular interactions underlying diseases demands the identification of biological units like transcription factors likewise the roadmap between different diseases. Considering the ever growing of omics data (15), data integration and analysis approaches are great keys for solving the disease’s complexities. In this study it was assumed that there is a sort of similarities among CD and HCV patients at the gene expression level. Therefore, networking was conducted to identify molecules underlying these disorders. The co-expressed genes that thought to have important influences in the pathogenesis of CD and HCV were then prioritized. Hereon, we attempted to provide a systematic understanding of the potential connections among these genes. 

## Methods

Firstly, curated gene-disease relations in CD and HCV was retrieved from DisGeNET v2.0 server (http://www.disgenet.org/web/DisGeNET). These sets has been collected from UNIPROT, human CTD, PsyGeNET, Orphanet and the HPO). Only disease-related genes with at least one evidence from pfam was selected for further analysis. Afterward, a list of 9905 regulatory links between human gene-transcription factors was obtained from TRRUST database (http://www.grnpedia.org/trrust/). These regulatory connections have been collected from 11,237 over 20 million PubMed articles and experimentally validated transcriptional regulations consisting of 821 human transcription factors and 2,159 non-TF genes. Sentence-based text mining approach has been employed for seeking these interactions. Next, for illustrating regulatory interactions among the genes, a regulatory network was built using the intersection of CD and HCV assigned genes and regulatory links derived by TRRUST. The transcriptional regulatory links were further visualized by Cytoscape 3.4.0 (http://www.cytoscape.org/). Genes within the network were separately ranked based on centrality statistics calculated by CytoNCA Cytoscape plugin (16). Pathway analysis was conducted on the inferred network using Reactom (http://reactome.org/). Additionally, GO biological terms were characterized by feeding the common genes in BINGO Cytoscape plugin (http://apps.cytoscape.org/apps/bingo). Finally, for elaborating the network of shared genes among CD-HCV with biological regulators like miRNAs and discovering the experimentally validated miRNAs which interact with these genes, experimentally validated gene-miRNA interactions were taken from miRWalk 2.0 server (http://zmf.umm.uni-heidelberg.de/apps/zmf/mirwalk2/miRpub.html) based on miRBase database. 

## Results

**Figure 1:**
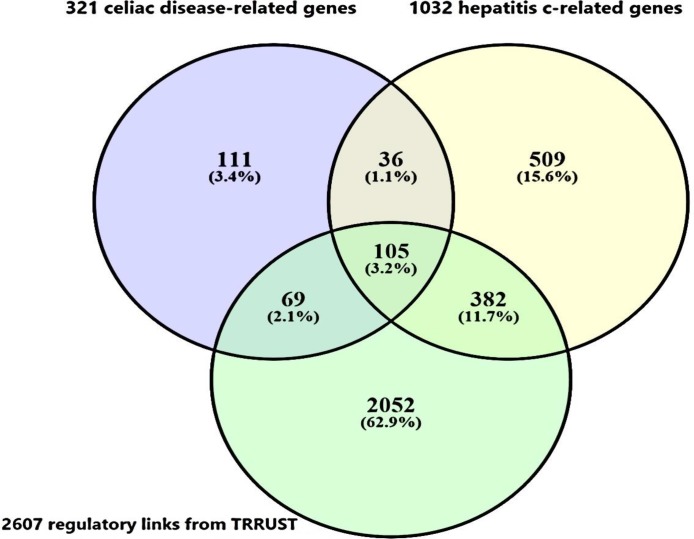
*Venn diagram of intersection between celiac disease, hepatitis c related genes retrieved from DisGeNET server and TRRUST regulatory links* *. Ultimately, 105 genes were taken as CD-HCV specific genes which are being in turn regulated by trascription factors based on the experimentally validated gene-TF links in TRRUST database*

**Figure 2:**
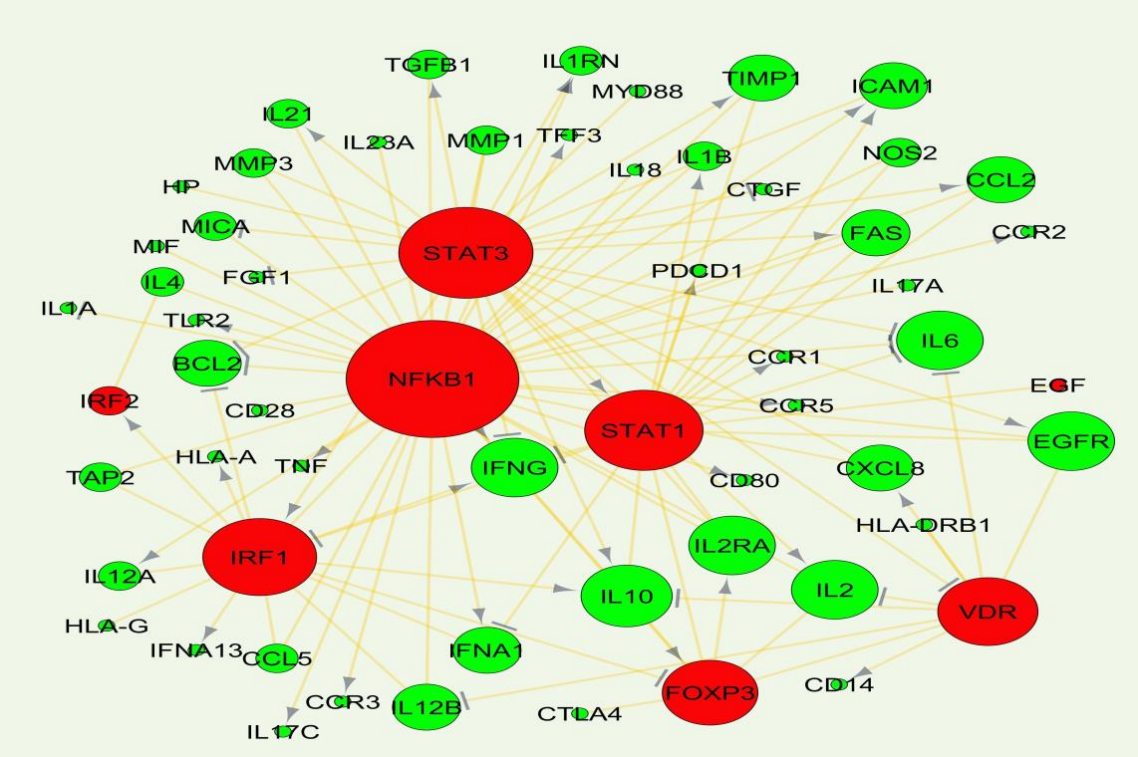
Interaction network established by common genes underlying celiac disease and hepatitis c. the green and red colors show genes and transcription factors respectively. Network units with more connectivity have been illustrated bigger.→ and ┴ arrows show the activation and repression effects of a TF on the targeted genes respectively

DisGeNET contains a list of diseases-associated genes collection based on the presence of genetic overlaps between diseases. In this list, among the 4,753,986 potential diseases associations, 13,064 diseases were found to share at least one gene. Among these disease-disease links, CD was significantly associated with arthritis, rheumatoid, atherosclerosis, multiple sclerosis and hepatitis C (combined score at P-value < 0.05) from which, CD and HCV were significantly related with a high combined score and then selected for mining the common underlying genes. Combined score is computed by taking the log of the p-value from the Fisher exact test and multiplying that by the z-score of the deviation from the expected rank. To this end, 321 and 1032 genes were retrieved from DisGeNET database, as CD and HCV specific genes respectively from which 105 gene were in common (figure 1). Among these 105 genes, CTNNB1, HNRNPD, NFKBIB, EGF, IRF1, IRF2, NFKB1,FOXP3, STAT1, STAT3 and VDR were extracted as transcription factors by intersecting the list of 105 genes and 821 human transcription factors-genes interactions derived from TRRUST database. Afterward, 2607 transcriptional relationships between these genes were exploited to establish a network (figure 2) by selecting the only highly ranked edges including EGF, IRF1, IRF2, NFKB1, FOXP3, STAT1, STAT3 and VDR transcription factors. 

**Figure 3:**
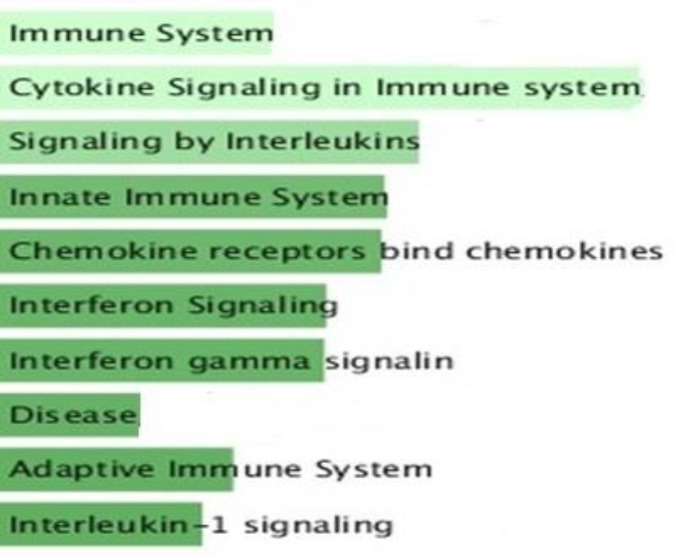
*Biological* *pathways* *in which* *common genes* *extracted from celiac disease and hepatitis c potentially involved by Reactom server with default parameters. The bars have been arranged top to down illustrating the number of genes and significance level assigned to each pathway*

In graph theory in addition to hub nodes (proteins with high degrees), certain nodes with higher centrality measures are more likely to be key connectors and likewise critical points controlling important dynamic components in biological networks (17) that removal of them causes biological systems fail to save their coherence. Hereby, in the generated network, NFKB1, STAT3, STAT1, IRF1, VDR and FOXP3 TFs, interleukin genes including IL10, IL6, IL2, IL2RA, epidermal growth factor receptor (EGFR), and interferon gamma (IFNG) showed the most connectivity. For adding a functional view to gene products within the regulatory network, functional classification was performed using BINGO Cytoscape plugin running hypergeometric test and Benjamini and Hochberg with False Discover Rate (FDR) correction at significant level 0.01 (figure 3). 

**Figure 4:**
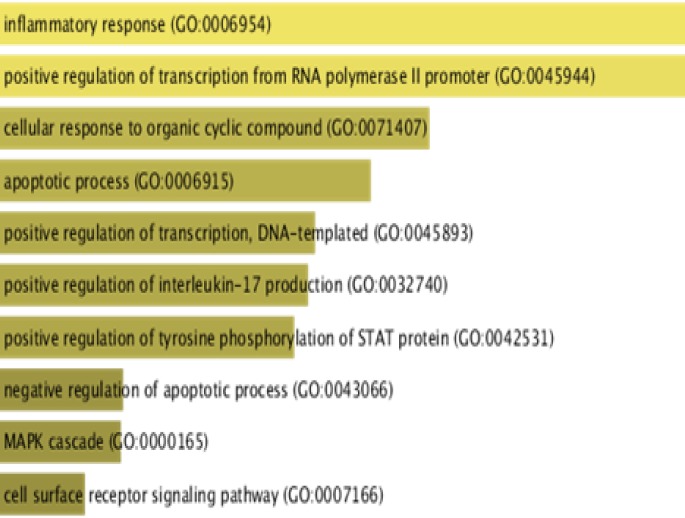
*Functional classification of biological processes in which differential expressed genes from common genes* *associated to celiac disease and hepatitis c* *supposed to be involved. The GO terms considered significant based on hypergeometric test with Benjamini and Hochberg FDR correction and significance level 0.01 by BINGO app.* *The bars have been arranged top to down illustrating the number of genes and significance level assigned to each GO term*

**Figure 5:**
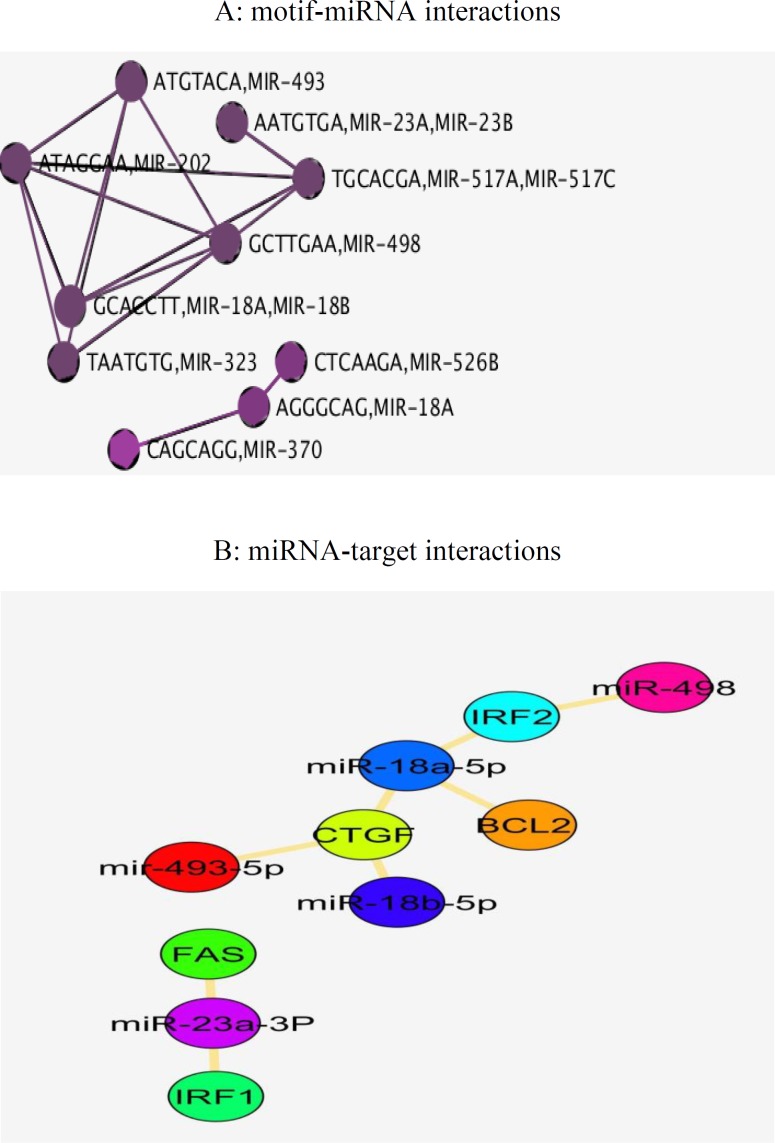
A, interacting miRNAs with the upstream motifs in common genes underlying celiac disease and hepatitis c retrieved from TargetScan. B, experimentally validated targets of explored miRNAs using miRWalk

According to the Figure 3, the genes were enriched in inflammatory response (STAT3, CLC2, FAS), apoptotic process (STAT1, CD14, IRF1) and MAPK cascade (EFGR, CCL5, CCR5, FGF1). Moreover in pathway analysis (figure 4), immune systems (innate and adaptive), mostly signaling pathways like chemokine, cytokine, interferon and interleukin1 were more noteworthy. Further study on the interplay in built network and microRNAs, was done by utilizing predicted miRNA-target networks from TargetScan (figure 5A) where miR-493, miR-23a/b miR-498, miR-498 and miR-18b were taken as the potential regulators and IRF1, FAS, IRF2, BCL2 and CTGF as their experimentally validated targets gathered from miRWalk (figure 5B).

## Discussion

The co-incidence of two or more disorders in an individual is named comfortability, therefore characterizing genes and pathways involved in the related diseases would be helpful in identifying comorbidity patterns (18). The potential connections of CD and HCV has been extensively investigated in a number of studies (19-23). It is noteworthy that HCV may activate immune response to gluten in people with genetic susceptibility (24) that could be a fair explanation in comorbidity of CD and HCV (25). Thereof, as HCV initiates autoimmune disease process and given the difficulty in determining interactions among the diseases, in the present study we employed freely available data and bioinformatics tools to investigate genes whose interactions is supposedly challenging in both of the CD and HCV pathogenesis and comorbidities. Among all possible 4,753,986 disease associations annotated in DisGeNET database, CD was significantly related with HCV with P-value < 0.05 and high combined score. Genetic overlap in gene ontology and pathway levels has been used to measure exploratory functions between two diseases in DisGeNET database. Since HCV as an exogenous cause of non-intestinal inflammatory responses is also able to induce immune systems (25), CD-HCV links was then chosen for investigating shared genetic components. Among the CD and HCV assigned genes, there are a number of interferon genes (IFNA1, IFNG) seemingly regulated by NFKB1, IFR1, STAT1 and STAT3 participating in inflammatory and apoptotic events (figures 2 and 3). Interestingly, interleukins were demonstrated as the most common genes regulated by all of the eight TFs in this study implying on indispensable role of interleukins in immune-mediated disorders. NFKB1 transcription factor with the highest centrality score within network, has the documented role in the regulation of interleukin genes (26). NFKB as a pivotal regulator of the adaptive immune system, modulates the activation, proliferation and survival of lymphocyte. NFKB pathway has been demonstrated to be constitutively upregulated in CD (27). In the core of NFKB signaling pathway, there are CD28 involved in proximal signaling, TNF, NOS2, STAT1, BCL and CLC all of them existed among the 105 common genes. As illustrated in the Figure 2, BLC2 is being suppressed by the most of transcription factors including VDR. Another aspect of scrutinized genes was the abundance of Human leukocyte antigen (HLA-A, HLA-G, HLA-DRB1) that are likely regulated by IRF1 and VDR in activating and unknown manners. HLA-mediated inflammatory reactions is thought to be linked with CD, HCV and other autoimmune disorders (28, 29) whereby a number of HLAs in this study were mainly regulated by IRF1 and VDR. IRF1 plays a role in triggering gluten intolerance that itself is being suppressed by STAT1 (figure 2). In this regard, FOXP3 is essential to regulating differentiation of T-cells in adaptive immunity (30) activated and suppressed by NFKB1 and IRF1 respectively. VDR are normally expressed in the duodenal mucosa of patients with CD, causes mucosal damage (31). HLAs as predisposing genetic factors contributes to the genetic pathogeny of CD at 70%-95% (32, 33). Finally, to demonstrate the implications of miRNAs in CD-HCV, we have identified miRNAs relevant with CD and HCV. In the presented study based on the experimentally validated gene-miRNA interactions collected from miRWalk, miR-23a-3P, miR-18a-5p, miR-493, miR-498-5p, miR-181/b-5p was found to regulated IRF1, FAS, IRF2, BCL2 and CTGF (figure 5). The dysregulation of miR-23a/b have been already documented to be involved in several of chronic inflammations (34). Among them FAS is being likely activated by STAT3 (figure 2). FAS was indicated to be associated with the severity of atrophy in CD (35). FAS also was found to mediate cell apoptosis by contributing with IL10, interferon genes, T-cell receptors (CD94) and TNF-α (36). This could give a partial picture of initiating malignant and nonmalignant proliferation in T-cells due to the comorbidity of CD and HCV. Among the 105 genes, IL23A and CD28 have shown altered expression in both of children and adult people with CD and while STAT3 only was differentially expressed in adult (37). The absurdness of interleukin genes is still noteworthy considering inflammatory response (GO: 0006954), cytokine signaling, chemokines, interferon, interleukin signaling pathways (figures 4, 5). Interleukins and interferon have proven roles in Subsets of immune and inflammatory events against autoimmune diseases like CD (38). Among the interleukin genes, IL6 is seemingly repressed by NFKB1, VDR, STAT3 and STAT1. IL6 has shown altered expression only in adult with CD. TNF, IL2 and IL21 were exhibited to induce proliferation of innate intraepithelial lymphocytes (39) that are being activated and suppressed by STAT3 and VDR transcription factors respectively. Viral products have been thought to have a potential to trigger the development of CD in genetically inclined individuals (40, 41), however the findings of CD-HCV associations are still controversial (42-46). Nevertheless of these studies, bioinformatics analysis potentials has been less used in addressing more important genes with similar expression patterns in CD and HCV. Taken together, several of genes and transcription factors were highlighted which thought to interact to each other in giving rise to CD-HCV comorbidity principally via immune responses. 

However this analysis included some of limitations; we generated an unweighted network, we then should note the dynamic nature of diseases via strictly analysis of static networks. Furthermore CD and HCV related genes were retrieved from adult and children forms of CD as well as all forms of HCV either acute, chronic and HCV-related hepatocellular carcinoma while CD can appear at any age but there are some differences in CD in children and adult form of CD. One therefore should be more specific to drive the exact types of disease-disease links using the strict criterion. Utilizing CD and HCV trade-off can help to explain disease inverse comorbidity. We noticed that CD-HCV specific genes were mainly assigned to pathways explicating the inflammation responses. Apparently the immune system reactions to each of them contribute to the activation in the setting of antibodies that in turn will cause of new symptoms even involved. Functional classification of common genes mainly in chemokine and cytokine pathways might be biologically relevant with the necessity of screening disease-associated antibodies prior to the therapies for declining inflammatory responses in these patients.

In conclusion, conserved genes and TFs obtained by network-based analysis like NFKB1, STAT3, IRF1, HLAs interleukin genes and chemokines could be proposed for future researches where their interactions could be proofed by confidently experiments.
